# Supplementary material for: Household food insecurity, maternal nutrition, environmental risks and infants’ health outcomes: protocol of the IMPALA birth cohort study in Uganda
Source: BMJ Open. 2022 Mar 18;12(3):e050729. doi: 10.1136/bmjopen-2021-050729 (PMC8935180; doi:10.1136/bmjopen-2021-050729)
Supplement: Author's manuscript [file bmjopen-2021-050729.draft_revisions.pdf]

BMJ Open

BMJ Open is committed to open peer review. As part of this commitment we make the peer review history of every article we publish publicly available.

When an article is published we post the peer reviewers' comments and the authors' responses online. We also post the versions of the paper that were used during peer review. These are the versions that the peer review comments apply to.

The versions of the paper that follow are the versions that were submitted during the peer review process. They are not the versions of record or the final published versions. They should not be cited or distributed as the published version of this manuscript.

BMJ Open is an open access journal and the full, final, typeset and author-corrected version of record of the manuscript is available on our site with no access controls, subscription charges or pay-per-view fees (http://bmjopen.bmj.com).

If you have any questions on BMJ Open's open peer review process please email info.bmjopen@bmj.com

BMJ Open

# Household food insecurity, maternal nutrition, environmental risks and infants' health outcomes: protocol of the IMPALA birth cohort study in Uganda

| | |
|---|---|
| Journal: | *BMJ Open* |
| Manuscript ID | bmjopen-2021-050729 |
| Article Type: | Protocol |
| Date Submitted by the Author: | 28-Feb-2021 |
| Complete List of Authors: | Terfa, Zelalem G.; Liverpool School of Tropical Medicine, Clinical Sciences ; Addis Ababa University, College of Business and Economics<br>Nantanda, Rebecca; Liverpool School of Tropical Medicine, Department of Clinical Sciences; Makerere University College of Health Sciences, Makerere University Lung Institute<br>Lesosky, Maia; University of Cape Town, Dept. of Public Health & Family Medicine; Liverpool School of Tropical Medicine, Department of Clinical Sciences<br>Devereux, Graham ; Liverpool School of Tropical Medicine, Department of Clinical Sciences<br>Obasi, Angela; Liverpool School of Tropical Medicine, Department of International Public Health<br>Mortimer, Kevin; Liverpool School of Tropical Medicine, Department of Clinical Sciences<br>Khan, Jahangir; Liverpool School of Tropical Medicine, Department of International Public Health; University of Gothenburg, School of Public Health and Community Medicine<br>Rylance, Jamie; Liverpool School of Tropical Medicine, Department of Clinical Sciences; Malawi-Liverpool-Wellcome Trust Clinical Research Programme, Lung Health Group<br>Niessen, Louis; Liverpool School of Tropical Medicine, Department of International Public Health ; Johns Hopkins University Bloomberg School of Public Health, Department of International Health |
| Keywords: | Respiratory infections < THORACIC MEDICINE, Economics < TROPICAL MEDICINE, Epidemiology < INFECTIOUS DISEASES |
| | |

SCHOLARONE™
Manuscripts

# Household food insecurity, maternal nutrition, environmental risks and infants' health outcomes: protocol of the IMPALA birth cohort study in Uganda

Zelalem G. Terfa[1, 3, *,‡], Rebecca Nantanda[1,4, *], Maia Lesosky[1,5], Graham Devereux[1], Angela Obasi[2], Kevin Mortimer[1], Jahangir Khan[2,6], Jamie Rylance[1,7, †], Louis W. Niessen[2,8, †], IMPALA Consortium

1. Department of Clinical Sciences, Liverpool School of Tropical Medicine, Liverpool, UK
2. Department of International Public Health, Liverpool School of Tropical Medicine, Liverpool, UK
3. College of Business and Economics, Addis Ababa University, Addis Ababa, Ethiopia
4. Makerere University Lung Institute, Makerere University College of Health Sciences, Kampala, Uganda
5. School of Public Health and Family Medicine, Faculty of Health Sciences, University of Cape Town, South Africa
6. School of Public Health and Community Medicine, University of Gothenburg, Sweden
7. Lung Health Group, Malawi Liverpool Wellcome Trust Clinical Research Programme, Queen Elizabeth Central Hospital, College of Medicine, P.O. BOX 30096, Chichiri, Blantyre, Malawi
8. Department of International Health, Johns Hopkins School of Public Health, Baltimore, Maryland, USA.

[*] equally contributed

[†] equally contributed

**Word count:** 3,633 words

[‡] Corresponding author: Email: Zelalem.Terfa@lstmed.ac.uk; Address: Pembroke Place, Liverpool L3 5QA UK



For peer review only

**Abstract**

**Introduction:** Food insecurity and undernutrition disproportionately affect women of reproductive age, infants and young children. The persistent disease burden from malnutrition in these vulnerable sections of societies remain a major concern in low-and middle-income countries (LMICs). Biomass fuel use for cooking is also common in LMICs. Empirical evidence on early life nutritional and environmental exposures and their effect on lung function of infants is important. Data from Sub-Saharan Africa are scarce.

**Aim:** To estimate the association between household food insecurity and energy poverty, maternal dietary diversity, and the lung function of infants.

**Methods and analysis:** Pregnant women will be recruited in an existing Health and Demographic Surveillance Site in South West Uganda. Household food insecurity and sources and uses of energy for cooking, economic measures and maternal dietary diversity will be collected during pregnancy. Follow-up data collection on the same variables will be repeated after birth. Primary health outcomes will be infant lung function determined by tidal breath flow and volume (TBFV) analysis at 6 -10 weeks of age.

A household food consumption score and minimum dietary diversity for women (MDD-W) indicator will be constructed. The involved cost of dietary diversity (CoDD) will be estimated based on MDD-W. The association between household level and mothers' food access indicators and lung function of infants will be evaluated using regression model. The multi-dimensional energy poverty index (MEPI) will be estimated and used as an indicator of households' environmental exposures. The association between household MEPI and lung function of infants will be assessed using econometric models.

**Ethics and dissemination:** Ethical approval to conduct the study has been obtained from Liverpool School of Tropical Medicine (research protocol (18-059)) and Uganda National Council for Science and Technology (SS 4846). Study results will be shared with policy makers and stakeholders and through publication in peer-reviewed journals.

**Study dates:** 1 April 2019 to 31 August 2021


For peer review only

**Strengths and limitations of this study**

- A key strength is the use of a birth cohort study design which allows prospective assessment of early life exposures and their association with infant lung function.
- We will be able to generate novel data with the potential to inform much needed interventions to address important global health challenges of nutrition and adverse environmental exposures.
- The main limitation is the necessity for clinic-based recruitment of pregnant women due to the availability of equipment to measure infant lung function which means that women who do not attend ANC will be systematically excluded. As these women are most likely to be disadvantaged and living in extreme poverty, this may represent an important selection bias.
- The fact that our study is located in only one district and that longstanding surveillance may have caused that district to be atypical in other ways. This could limit the generalisability of our findings.

For peer review only


For peer review only - http://bmjopen.bmj.com/site/about/guidelines.xhtml

## Introduction

Over the past two decades, the world has witnessed significant progress in poverty reduction. Despite the achievements made in reducing poverty across the globe, food insecurity and undernourishment are still pervasive in Sub-Saharan Africa (SSA). Data shows a fluctuating trend in progress made in the SSA region. The proportion of undernourished people in SSA declined from 28.1% in 2000 to 20.6% in 2010, remaining constant until 2015, and then increased to 22.8% in 2018 [1 2]. In the SSA region, Eastern Africa has the highest prevalence (30.8%) of undernourishment with more than 133 million affected [2]. Prevalence of total food insecurity is also highest in East Africa, increasing to 62.7 % in 2018 from 58.2% in 2014 [2]. In Uganda specifically the proportion of undernourished population increased to 39% between 2014 and 2016 after showing a declining trend between 2004 and 2006 [1].

Food insecurity and malnutrition disproportionately affect women of reproductive age, infants and young children in low- and middle-income countries (LMIC)[3-6]. Women living in LMICs are particularly at risk of malnutrition during pregnancy due to socio-economic constraints, high intensity of agricultural labour, and short interpregnancy intervals [7]. The high physiological demands of pregnancy and lactation also increase dietary requirements.

A child's nutritional status is inextricably linked to their mother's nutrition from pregnancy through the postpartum period [8]. Intergenerational effects increase infant mortality and have longer-term sequelae [4 9 10]. A study in Tanzania reported that about 40% of mothers with children below 24 months old did not consume a diverse diet and 35% of children were stunted [11]. Household food insecurity is a risk factor for iron-deficiency anaemia in infants and toddlers [12], is associated with small birth size [13], and adverse health outcomes among infants [14]. Early life nutritional exposures, combined with changes in lifestyle in adult life, can result in increased risk of chronic diseases in later life [8].

Poorer households tend to select foods of lower nutritional value and lower-quality diets; this is particularly true in low income countries [15 16]. Household-level strategies to cope with any shortfall in food availability tend to further reduce both quality and quantity [17], and food prices can significantly moderate this effect [18]. Rises in the price of food also lead to reduction in dietary diversity [19] and consequently nutritional outcomes [16].

Household air pollution exposure is a predisposing factor for adverse health outcomes in children. In higher income countries, pre-natal exposure to air pollution is associated with higher minute ventilation and tidal flows and airway inflammation in new-borns [20]. Exposure to biomass smoke was associated with deficits in lung function in adults in Nepal [21]. A study in India showed that poverty is strongly associated with reduced paediatric lung function, in addition to body mass index, tobacco exposure and biomass use [22]. A recent study in rural Ghana also showed that prenatal household air pollution exposure is associated with impaired infant lung function [23]. Household energy poverty index , a measure of households deprivation from clean energy source [24], could be used as a proxy to assess the association between household air pollution and lung health outcome of young infants.



For peer review only - http://bmjopen.bmj.com/site/about/guidelines.xhtml

For peer review only

Empirical evidence on the association of early life nutritional and environmental exposures with infants' lung functions and general health outcomes is important to help policy makers in setting priorities to improve early childhood development and identify intervention mechanisms. Empirical evidence on the effects of nutritional exposures and health outcome of infants are largely from higher income countries. Studies from some LMICs focusing on infants' nutritional exposure in the womb are scarce. No studies from Sub-Saharan Africa have examined the effect of households' nutritional and environmental exposure on the health outcome of infants, particularly the lung functions of infants and mothers. Inequalities in lung health outcomes of infants across socioeconomic status of households is also unknown in the region. We will generate unique quantitative evidence on prevalence and distribution of food insecurity, dietary diversity among pregnant women and postnatal mothers together with cost of dietary diversity (CoDD), energy poverty and its association with lung function and other general health outcomes in infants in low-income settings.

**Study goal and objectives**

The study aims to estimate the association of households' food insecurity, mothers' dietary diversity, and energy poverty with infant lung function and general health outcomes. The specific objectives are as follows:

1. To estimate food insecurity at household level, dietary diversity of pregnant women, and related cost of dietary diversity (CoDD).
2. To assess the effect of household food insecurity and women's dietary diversity during pregnancy and after birth on infant's lung function and general health outcomes.
3. To estimate the association between the household energy poverty of pregnant women and their socio-economic status with the lung function of infants and mothers.

**Methods**

**Study design**

The study will use a prospective cohort study to measure exposure and outcomes among pregnant women and their infants. Data on household food consumption and energy sources, and maternal dietary diversity will be collected during pregnancy and 6 to 10 weeks after birth. Infants' lung function and other health outcome data will also be collected at 6-10 weeks after delivery.

**Study setting**

This study, which is part of a larger, multi-country research programme, the International Multidisciplinary Programme to Address Lung Health and TB in Africa (IMPALA) [25]. The study will be conducted in the General Population Cohort (GPC) which is a Health and Demographic Surveillance Site (HDSS) in Kyamulibwa sub-county of Kalungu District in


For peer review only - http://bmjopen.bmj.com/site/about/guidelines.xhtml

South-Western Uganda. The GPC is a population-based open cohort study, established in 1989 by the UK Medical Research Council (MRC) in collaboration with the Uganda Virus Research Institute (UVRI) primarily to examine trends in the prevalence and incidence of HIV and their determinants in rural south-Western Uganda[26] . The HDSS comprises 25 rural villages, and a small township of Kyamulibwa Town Council. Annual census and medical surveys have been conducted in this population since 1989 [27] .

The main economic activity in the site is agriculture characterized by small farms of bananas, coffee, legumes, vegetables and a few root crops such as cassava and potatoes. There are also small-scale cattle, goat and pig farming. A few of the residents are engaged in small scale trade mainly selling coffee, food crops and fish. The education level of inhabitants is generally low with only one third attaining secondary level schooling. There is no special health, economic and social support programmes in the HDSS. Therefore, the livelihoods and challenges of the residents are generally like that of people in other rural parts of Uganda.

Five health facilities serve the population with basic medical care, of which three facilities offer maternity services, antenatal care deliveries and postnatal care. The HDSS has a pregnancy and birth registration system where pregnant women are registered and followed up until delivery to document birth outcomes. Births are notified to the HDSS administration within 24-48 hours. In Uganda, 97% of women of reproductive age receive at least one antenatal care (ANC) [28]. The existing systems within the HDSS are favourable for conducting cohort studies, including the established birth notification and registration system as they run periodic census and medical surveys. This would make it easier to track mothers through pregnancy and delivery of their babies, and follow them up for lung function testing, thus minimizing loss to follow up, and allowing future studies.

**Study population**

The study will recruit pregnant women, their infants and households.

**Sampling strategy**

This cohort study will be conducted at the three health facilities that offer maternity services in the HDSS. Pregnant women will be identified through the antenatal clinics, where they will be enrolled consecutively. All pregnant women will be approached to participate in the study. They will be provided with an information sheet in the local language to read whilst waiting to be seen. If necessary, the clinic staff will read the information sheet and explain the study to the potential participants. After the clinic consultation the clinician will ascertain if the potential participant is interested. Participants who express an interest in taking part will be directed to the research team at the end of the clinic appointment. Informed written consent will then be obtained.

Same day recruitment will be performed where possible to minimise inconvenience and costs of repeat travel for participants. However, if potential participants express a wish to take time to think things over and return another day, this will be facilitated.

For peer review only

**Sample size calculation**

The sample size estimation is based on estimation of the association between the primary exposures (maternal dietary diversity and household food security) with infant lung function. The primary outcome will be infant lung function and study power is based on a birth cohort study on air pollution during pregnancy and lung function in infants in which air pollution levels were related with the lung function in infants aged 6-10 weeks, and reported a mean (SD) of 1401 (242) ml/l [20].

Maternal dietary diversity is measured by a dichotomous indicator while household food insecurity is a three-scale categorical variable. The proposed study will have 80% power to detect a 5% difference in minute volume between high and low maternal dietary diversity groups and a 6% difference across the thirds of household food insecurity at the 5% level of significance. With a minimum sample size of 360 (complete data), we will have at least 80% power to estimate moderate effect sizes (OR = 1.5 or larger) under a logistic regression model, and >90% power to estimate differences of 5% in continuous infant lung function outcomes with 95% confidence, under a linear regression model. We therefore expect to recruit 560 pregnant women to account for an anticipated 10% loss to follow up, a 5% rate of miscarriages, still births, and neonatal deaths, and for an unattainable measurement of primary outcome in 25%.

**Eligibility criteria**

*Pregnant women:* All pregnant women presenting for ANC services at the selected health facilities and residing within the HDSS will be eligible for participation in the study. Women who will not be available throughout the entire study period (such as those intending to go to another area for delivery of their babies or other reasons) and/or unwilling to participate in the study will be excluded.

*Infants:* All singleton infants will be eligible. It will not be possible to conduct lung function tests for infants with congenital abnormalities of the upper airway because of the difficulties associated with interpreting the lung function findings from such infants. Therefore, these infants will be excluded at analysis of the main outcome (lung function).

**Data collection and analysis methods**

The data collection process will follow a number of steps. The data will be collected at four-time points; 1) Time point 1:during the antenatal clinic, recruitment point; 2) Time point 2: home visit to the pregnant woman households; 3) time point 3: lung function testing for mothers and their new baby, 6 to 10 weeks after birth, and 4) Time point 4: home visit during 6 to 10 weeks after birth. Summary of the data collection process and required data at each



For peer review only - http://bmjopen.bmj.com/site/about/guidelines.xhtml

of the four-time points is presented in flowchart (Figure 1). Details of data collection and analytical methods are presented under each of the sub-study topics. There will be three sub-studies as listed below.

**(Figure 1 about here!)**

**Study 1: Estimate food insecurity at household level, dietary diversity of pregnant women together with cost of dietary diversity (CoDD)**

The main research interest under this component of the study is to characterize the households of pregnant women by food insecurity and dietary diversity using baseline data. Closely related to dietary diversity of pregnant women, the cost of dietary diversity (CoDD) will be estimated. Socioeconomic determinants of household food insecurity and women's dietary diversity will also be identified.

**Data collection**

Household level food consumption and expenditure data will be collected using the World Bank's living standard questionnaire that has been used in many African countries, including Uganda. Consumption questionnaire will be mapped with Food consumption score (FCS) food items and groups. The food consumption score (FCS) is a validated tool to assess household level food insecurity. It combines data on dietary diversity and food frequency using 7 days recall data [29]. The main advantage of this tool is that it captures dietary diversity and household food access. It also enables an understanding of the usual consumption behaviour of households [30 31]. Therefore, FCS will be used to assess household level food insecurity during pregnancy (and after birth). Data on household's food insecurity coping strategies will also be collected. In addition, separate data will be collected using the Minimum Dietary Diversity for Women(MDD-W)questionnaire, a validated tool, [3] to assess pregnant women's dietary diversity. We will do adaptation work for the MDD-W questionnaire based on local context. Adaptation of the MDD-W questionnaire is helpful for adequate listing of locally available food items, contents of mixed food and food items that are consumed in trivial amounts and to reflect cultural norms, vocabulary and usage of words and phrases that will be easily understood.

A nationally representative monthly food price data will be obtained from Uganda Bureau of Statistics to estimate the cost of dietary diversity (CoDD) for women of reproductive age. CoDD is a price index defined around the MDD-W. It is an indicator that provides the least expensive way of meeting the MDD-W [32].

We shall collect anthropometric data (weight, height, and Mid Upper Arm Circumference-MUAC) from pregnant women. The same anthropometric measurement equipment will be used across the clinics and in all selected households. As described above women's anthropometric measurements will be taken at clinics during pregnancy check-up visits. Data on household asset ownership will be collected. These data will include education, access to


For peer review only - http://bmjopen.bmj.com/site/about/guidelines.xhtml

land, livestock holdings, type of crops grown, farm and other household assets, non-farm activities of the household. This data will be used to construct household asset index.

**Data Analysis:** Data will be analysed using both descriptive and econometric techniques. The household level food consumption score (FCS) will be obtained from frequency of consumption of each food group in the household and by assigning weight for each group. This score will be used to categorize households into three levels of food insecurity status; poor, borderline, and acceptable [29 30 33 34]. To construct the MDD-W indicator, food groups and sub-food groups will be aggregated into 10 MDD-W food groups. Woman's dietary diversity will be categorised into two based on standard threshold: scoring ≥ 5 and below the threshold [3 35 36]. The CoDD will be estimated based on rank order optimization based on food prices within group following Masters, et al. [32]. In this technique, cheapest food items will be selected from each of the ten food groups then the five most cheapest food items will be selected. This identifies the cheapest way to achieve MDD-W. This will be done on monthly basis and will be linked with monthly dietary diversity of pregnant women.

Associations of household level food insecurity and women's dietary diversity will be assessed using parametric or non-parametric statistical tests depending on distribution of the variables. Determinants of household food insecurity, and dietary diversity amongst women will be assessed using discrete choice based econometric models. The household level food insecurity, the dependent variable, is an ordered categorical variable. This suggests the natural choice will be the ordered logit (or probit) econometric model. Mother's dietary diversity takes dichotomous variables. Therefore, a logit (or probit) econometric model will be used to identify determinants of dietary diversity in women. Principal Component Analysis (PCA) will be used to construct households socioeconomic status index, using asset data, to categorize households into socioeconomic quintiles and the method is explained elsewhere [37 38]. Then distribution of household food insecurity and women's dietary diversity across socioeconomic status will be assessed.

**Study 2: Assessing the effect of household food insecurity and women's dietary diversity on infant's lung function and general health outcomes**

The dynamics of household food insecurity during pregnancy and after birth and its impacts on lung function and the general health outcomes measures (birth weight and length) of infants will be assessed. Changes in mothers' dietary diversity during pregnancy and after birth will be thoroughly assessed.

**Data collection**

Data collection from study 1 will be repeated in the same individuals 6 to 10 weeks after birth. In addition, data on health outcome of infants will be collected. This will include lung function, weight and length of infants. Data on lung function and anthropometric data (weight and hight) for mothers will also be collected. Lung function testing in the infants will be done using Tidal Breath Analysis (TBA) method by clinical sciences research team in IMPALA programme. From this analysis, tidal breathing measures of tidal volume (VT), respiratory rate (RR) and expiratory flow ratios of infants will be collected.


For peer review only - http://bmjopen.bmj.com/site/about/guidelines.xhtml

## Data Analysis

Household level food insecurity, and mother's diversity will be measured using the techniques discussed under study 1. The dynamics in household food insecurity and women's dietary will be analysed using descriptive statistics.

The association between household level and mothers' food access indicators and lung functions (measured by using MV, RR, tPEF:tE) and other health outcomes (weight-for-age and length-for-age) of infants will be evaluated using appropriate econometric models. We propose to use multiple linear regression model to estimate the association.

Household socioeconomic status will be categorized based on asset index. Principal Component Analysis (PCA) will be used to construct household socioeconomic status index. Distributions of lung function and other health outcome across socioeconomic status of households will be examined using a concentration curve [39 40]. The concentration curve gives a more complete picture of socioeconomic inequality in health outcomes. However, it does not give a measure of the magnitude of inequality [37]. Therefore, concentration index, which quantifies the degree of socioeconomic related inequality in a health variable [39-41] will be used to measure the degree of socioeconomic-related inequality in infant's and mother's lung function and other health outcomes.

These analyses will lead us to identify socioeconomic determinants of infants' lung function and other health outcome (nutritional status of infants).

**Study 3: Estimate the association of household energy poverty and their socio-economic status with lung function of infants and mothers**

This part of the study aims to assess association between household energy poverty and lung function of infants and their mothers. The sources of energy used by households, available/alternative energy sources, and households' access to alternative energy sources and implication for risk of respiratory diseases will be explored. Inequalities in health outcome (lung function) of infants across socioeconomic status of households will be explored.

## Data collection

Data on household energy source and use will be collected. Attention will be given to energy sources used by households, access to different sources of energy, energy use by category of use. We will also collect data on ownership of appliances, entertainment and communication equipment that rely on access to electricity. Lung function of infants and their mothers will be measured at 6 to 10 weeks of age using Tidal Breath Analysis method as described under study-2.

## Data analysis

Multi-dimensional energy poverty index (MEPI) will be estimated from household energy use and sources data. The MEPI captures and evaluate a set of energy deprivations that affect



For peer review only - http://bmjopen.bmj.com/site/about/guidelines.xhtml

households. Following Nussbaumer, et al. [24], the MEPI that is composed of five dimensions representing basic energy services and six indicators of these dimensions will be estimated. Essentially, a household is identified as energy poor if the respective set of deprivation exceeds a pre-defined threshold. However, there is no standard and agreed threshold. We will categorize households into quintiles based on MEPI and assess the distribution of infants' and women's lung function along the quintiles. Inequalities in health outcome of infants will be assess using method discussed under study-2. Association between energy poverty and infants' lung function will be evaluated using econometric models.

**Patient and public involvement**

We appreciate movement towards involvement of patients and the public as co-producers of research. Unfortunately, patients were not directly involved in the development of this research project. We will seriously consider this in future studies.

**Discussion**

The study will provide new and valuable quantitative empirical evidence on the relationships and interactions between household level food insecurity, mothers dietary diversity, energy poverty, and infants' lung function and general health outcomes in infants in low-income settings**.** We are not aware of other any similar studies in Sub-Sahara Africa. This evidence is critical for policy makers to make informed decisions in identifying and prioritizing interventions to address the burden of maternal malnutrition during pregnancy. It also provides a better understanding of public health implications of energy poverty and indoor air pollution at household level.

A key strength of this study is the use of a birth cohort study design which allows prospective assessment of early life exposures and their association with infant health outcomes. The necessity for clinic-based recruitment of pregnant women due to the availability of equipment to measure infant lung function, means that women who do not attend ANC will be systematically excluded. As these women are most likely to be disadvantaged and living in extreme poverty, this may represent an important selection bias. However, ANC uptake in Uganda is high [28] which should limit the size of this bias. The fact that our study is located in only one district and that longstanding surveillance [42] may have caused that district to be atypical in other ways, could limit the generalisability of our findings.

However, improving infant nutrition is a key global health priority. It is also increasingly recognised that poor lung function may be associated with increased morbidity across the life course [42]. Our prospective study will generate novel data with the potential to inform much needed interventions to address these important global health challenges.

**Figure legend/caption:**

**Figure 1** Flowchart for summary of data collection process and required data at each of data collection steps.



For peer review only - http://bmjopen.bmj.com/site/about/guidelines.xhtml

**Ethics and dissemination**

The study received ethical approval from Liverpool School of Tropical Medicine (LSTM) (research protocol (18-059)) and Uganda National Council for Science and Technology (SS 4846). Findings from this study will be disseminated through peer-reviewed publications and conference presentations. We also intend to disseminate the findings using other platforms including community feedback meetings and engagement with national policy makers and health care planners.

**Funding:** This research was commissioned by the National Institute of Health Research using Official Development Assistance (ODA) funding (IMPALA grant number 16/136/35). The views expressed in this publication are those of the author(s) and not necessarily those of the NHS, the National Institute for Health Research or the Department of Health.

**First and senior Authors:** ZGT and RN equally contributed and are joint first authors. LWN and JR are joint senior authors

**Contributors:** ZGT, RN, JR, JK, LWN conceptualized and designed the study. ZGT and RN wrote the protocol and drafted the protocol manuscript with guidance from LWN, JR, and JK. GD helped during protocol development and contributed in methodological aspect of the study. ML helped with methodological aspect of the study, including sampling design and sample calculations. AO critically reviewed the protocol manuscript and helped during initial stage of designing the study. KM is director of IMPALA and played key role during early conceptualization of the study. IMPALA consortium members provided useful comments to protocol manuscript.

**Collaborators:** On behalf of The NIHR International Multidisciplinary Programme to Address Lung Health and TB in Africa (IMPALA) Consortium, which in addition to the above named authors comprises: Emmanuel Addo-Yobo, Brian Allwood, Hastings Banda, Imelda Bates, Amsalu Binegdie, Asma El Sony, Adegoke Falade, Bertrand Mbatchou, Hellen Meme, Beatrice Mutayoba, Muhwa Jeremiah Chakaya, Nyanda Elias Ntinginya, S Bertel Squire, Miriam Taegtmeyer, Rachel Tolhurst, William Worodria, Heather Zar, Eliya Zulu, Lindsay Zurba.


For peer review only - http://bmjopen.bmj.com/site/about/guidelines.xhtml

## References


1. FAO. Regional Overview of Food Security and Nutrition in Africa 2017. The food security and nutrition–conflict nexus: building resiliencefor food security, nutrition and peace. Accra, 2017.
2. FAO, IFAD, UNICEF, et al. The State of Food Security and Nutrition in the World 2019. Safeguarding against economic slowdowns and downturns. Rome, FAO., 2019.
3. FAO, FHI 360. Minimum Dietary Diversity for Women: A Guide for Measurement. Rome:: FAO, 2016.
4. Lartey A. Maternal and child nutrition in Sub-Saharan Africa: challenges and interventions. *Proceedings of the Nutrition Society* 2008;67(1):105-08. doi: 10.1017/S0029665108006083 [published Online First: 2008/01/30]
5. Allen LH. Multiple micronutrients in pregnancy and lactation: an overview. *The American Journal of Clinical Nutrition* 2005;81(5):1206S-12S. doi: 10.1093/ajcn/81.5.1206
6. Black RE, Allen LH, Bhutta ZA, et al. Maternal and child undernutrition: global and regional exposures and health consequences. *The Lancet* 2008;371(9608):243-60. doi: https://doi.org/10.1016/S0140-6736(07)61690-0
7. Lee SE, Talegawkar SA, Merialdi M, et al. Dietary intakes of women during pregnancy in low- and middle-income countries. *Public Health Nutrition* 2013;16(8):1340-53. doi: 10.1017/S1368980012004417 [published Online First: 2012/10/09]
8. Christian P, Stewart CP. Maternal Micronutrient Deficiency, Fetal Development, and the Risk of Chronic Disease. *The Journal of Nutrition* 2010;140(3):437-45. doi: 10.3945/jn.109.116327
9. Roseboom TJ, van der Meulen JHP, Ravelli ACJ, et al. Effects of prenatal exposure to the Dutch famine on adult disease in later life: an overview. *Molecular and Cellular Endocrinology* 2001;185(1):93-98. doi: https://doi.org/10.1016/S0303-7207(01)00721-3
10. Victora CG, Adair L, Fall C, et al. Maternal and child undernutrition: consequences for adult health and human capital. *The Lancet* 2008;371(9609):340-57. doi: 10.1016/S0140-6736(07)61692-4
11. Huang M, Sudfeld C, Ismail A, et al. Maternal Dietary Diversity and Growth of Children Under 24 Months of Age in Rural Dodoma, Tanzania. *Food and Nutrition Bulletin* 2018:0379572118761682. doi: 10.1177/0379572118761682
12. Park K, Kersey M, Geppert J, et al. Household food insecurity is a risk factor for iron-deficiency anaemia in a multi-ethnic, low-income sample of infants and toddlers. *Public Health Nutrition* 2009;12(11):2120-28. doi: 10.1017/S1368980009005540 [published Online First: 2009/05/01]
13. Chowdhury M, Dibley MJ, Alam A, et al. Household Food Security and Birth Size of Infants: Analysis of the Bangladesh Demographic and Health Survey 2011. *Current Developments in Nutrition* 2018;2(3):nzy003-nzy03. doi: 10.1093/cdn/nzy003
14. Cook JT, Frank DA, Berkowitz C, et al. Food Insecurity Is Associated with Adverse Health Outcomes among Human Infants and Toddlers. *The Journal of Nutrition* 2004;134(6):1432-38. doi: 10.1093/jn/134.6.1432
15. Darmon N, Drewnowski A. Contribution of food prices and diet cost to socioeconomic disparities in diet quality and health: a systematic review and analysis. *Nutrition Reviews* 2015;73(10):643-60. doi: 10.1093/nutrit/nuv027
16. Miller V, Yusuf S, Chow CK, et al. Availability, affordability, and consumption of fruits and vegetables in 18 countries across income levels: findings from the Prospective Urban Rural Epidemiology (PURE) study. *The Lancet Global Health* 2016;4(10):e695-e703. doi: https://doi.org/10.1016/S2214-109X(16)30186-3
17. Farzana FD, Rahman AS, Sultana S, et al. Coping strategies related to food insecurity at the household level in Bangladesh. *PLOS ONE* 2017;12(4):e0171411. doi: 10.1371/journal.pone.0171411




For peer review only - http://bmjopen.bmj.com/site/about/guidelines.xhtml

18. Green R, Cornelsen L, Dangour AD, et al. The effect of rising food prices on food consumption: systematic review with meta-regression. *BMJ : British Medical Journal* 2013;346:f3703. doi: 10.1136/bmj.f3703
19. Brinkman H-J, de Pee S, Sanogo I, et al. High Food Prices and the Global Financial Crisis Have Reduced Access to Nutritious Food and Worsened Nutritional Status and Health. *The Journal of Nutrition* 2009;140(1):153S-61S. doi: 10.3945/jn.109.110767
20. Latzin P, Roosli M, Huss A, et al. Air pollution during pregnancy and lung function in newborns: a birth cohort study. *The European respiratory journal* 2009;33(3):594-603. doi: 10.1183/09031936.00084008 [published Online First: 2008/11/18]
21. Kurmi OP, Devereux GS, Smith WCS, et al. Reduced lung function due to biomass smoke exposure in young adults in rural Nepal. *European Respiratory Journal* 2013;41(1):25-30. doi: 10.1183/09031936.00220511
22. Sonnappa S, Lum S, Kirkby J, et al. Disparities in Pulmonary Function in Healthy Children across the Indian Urban–Rural Continuum. *American Journal of Respiratory and Critical Care Medicine* 2015;191(1):79-86. doi: 10.1164/rccm.201406-1049OC
23. Lee AG, Kaali S, Quinn A, et al. Prenatal Household Air Pollution Is Associated with Impaired Infant Lung Function with Sex-Specific Effects. Evidence from GRAPHS, a Cluster Randomized Cookstove Intervention Trial. *Am J Respir Crit Care Med* 2019;199(6):738-46. doi: 10.1164/rccm.201804-0694OC [published Online First: 2018/09/27]
24. Nussbaumer P, Bazilian M, Modi V. Measuring energy poverty: Focusing on what matters. *Renewable and Sustainable Energy Reviews* 2012;16(1):231-43. doi: https://doi.org/10.1016/j.rser.2011.07.150
25. Ding Y, Pulford J, Bates I. Practical actions for fostering cross-disciplinary global health research: lessons from a narrative literature review. *BMJ Global Health* 2020;5(4):e002293. doi: 10.1136/bmjgh-2020-002293
26. Asiki G, on behalf of the GPCt, Murphy G, et al. The general population cohort in rural south-western Uganda: a platform for communicable and non-communicable disease studies. *International Journal of Epidemiology* 2013;42(1):129-41. doi: 10.1093/ije/dys234
27. Asiki G, Murphy GAV, Baisley K, et al. Prevalence of Dyslipidaemia and Associated Risk Factors in a Rural Population in South-Western Uganda: A Community Based Survey. *PLOS ONE* 2015;10(5):e0126166. doi: 10.1371/journal.pone.0126166
28. UBOS, ICF. Uganda Bureau of Statistics (UBOS) and ICF. 2018. Uganda Demographic and Health Survey 2016. Kampala, Uganda and Rockville, Maryland, USA: UBOS and ICF., 2018.
29. WFP. World Food Programme. Food consumption analysis: calculation and use of the Food Consumption Score in food consumption and food security analysis. Technical Guidance Sheet. Rome. 2008
30. Jones AD, Ngure FM, Pelto G, et al. What Are We Assessing When We Measure Food Security? A Compendium and Review of Current Metrics. *Advances in Nutrition* 2013;4(5):481-505. doi: 10.3945/an.113.004119
31. Maxwell D, Vaitla B, Coates J. How do indicators of household food insecurity measure up? An empirical comparison from Ethiopia. *Food Pol* 2014;47:107-16. doi: https://doi.org/10.1016/j.foodpol.2014.04.003
32. Masters WA, Bai Y, Herforth A, et al. Measuring the Affordability of Nutritious Diets in Africa: Price Indexes for Diet Diversity and the Cost of Nutrient Adequacy. *Amer J Agr Econ* 2018;100(5):1285-301. doi: 10.1093/ajae/aay059
33. Wiesmann D, Bassett L, Benson T, et al. Validation of the World Food Programme s Food Consumption Score and Alternative Indicators of Household Food Security: Intl Food Policy Res Inst 2009.
34. Leroy JL, Ruel M, Frongillo EA, et al. Measuring the Food Access Dimension of Food Security:A Critical Review and Mapping of Indicators. *Food and Nutrition Bulletin* 2015;36(2):167-95. doi: 10.1177/0379572115587274

For peer review only

35. Arimond M, Wiesmann D, Becquey E, et al. Simple Food Group Diversity Indicators Predict Micronutrient Adequacy of Women's Diets in 5 Diverse, Resource-Poor Settings. *The Journal of Nutrition* 2010;140(11):2059S-69S. doi: 10.3945/jn.110.123414
36. Saha KK, Frongillo EA, Alam DS, et al. Household food security is associated with growth of infants and young children in rural Bangladesh. *Public Health Nutrition* 2009;12(9):1556-62. doi: 10.1017/S1368980009004765 [published Online First: 2009/09/01]
37. O'Donnell O, van Doorslaer E, Wagstaff A, et al. Analyzing health equity using household survey data: a guide to techniques and their implementation. Washington, D.C: World Bank Publications 2008.
38. Vyas S, Kumaranayake L. Constructing socio-economic status indices: how to use principal components analysis. *Health Policy and Planning* 2006;21(6):459-68. doi: 10.1093/heapol/czl029
39. Kakwani N, Wagstaff A, van Doorslaer E. Socioeconomic inequalities in health: Measurement, computation, and statistical inference. *J Econometrics* 1997;77(1):87-103. doi: https://doi.org/10.1016/S0304-4076(96)01807-6
40. Wagstaff A, Paci P, van Doorslaer E. On the measurement of inequalities in health. *Social Science & Medicine* 1991;33(5):545-57. doi: https://doi.org/10.1016/0277-9536(91)90212-U
41. Wagstaff A, van Doorslaer E, Watanabe N. On decomposing the causes of health sector inequalities with an application to malnutrition inequalities in Vietnam. *J Econometrics* 2003;112(1):207-23. doi: https://doi.org/10.1016/S0304-4076(02)00161-6
42. Agustí A, Noell G, Brugada J, et al. Lung function in early adulthood and health in later life: a transgenerational cohort analysis. *Lancet Respir Med* 2017;5(12):935-45. doi: 10.1016/s2213-2600(17)30434-4 [published Online First: 2017/11/19]

For peer review only

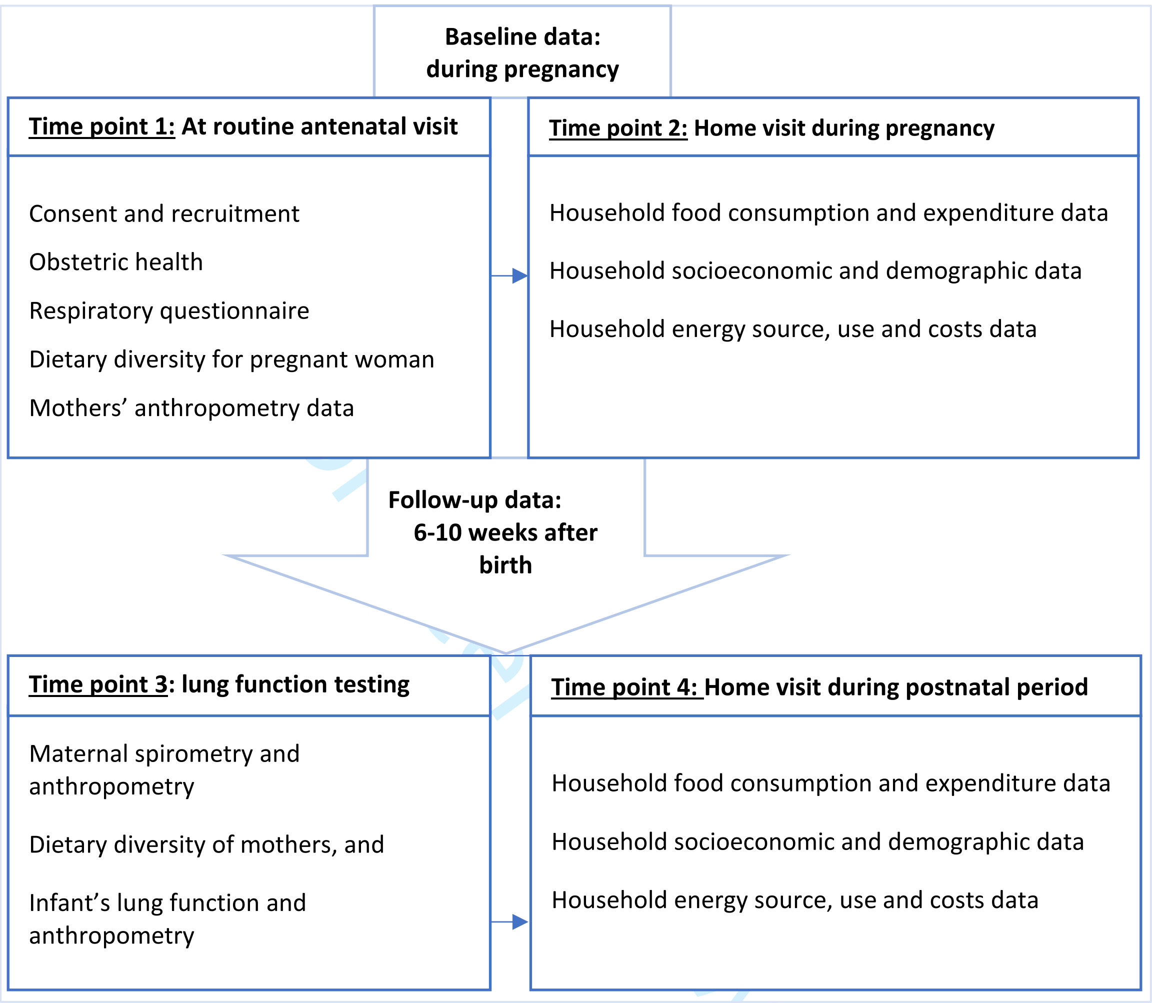


**Figure 1** Flowchart for summary of data collection process and required data at each of data collection steps.

# Household food insecurity, maternal nutrition, environmental risks and infants' health outcomes: protocol of the IMPALA birth cohort study in Uganda


| | |
|---|---|
| Journal: | *BMJ Open* |
| Manuscript ID | bmjopen-2021-050729.R1 |
| Article Type: | Protocol |
| Date Submitted by the Author: | 09-Dec-2021 |


| | |
|---|---|
| Complete List of Authors: | Terfa, Zelalem G.; Liverpool School of Tropical Medicine, Clinical Sciences ; Addis Ababa University, College of Business and Economics<br>Nantanda, Rebecca; Liverpool School of Tropical Medicine, Department of Clinical Sciences; Makerere University College of Health Sciences, Makerere University Lung Institute<br>Lesosky, Maia; University of Cape Town, Dept. of Public Health & Family Medicine; Liverpool School of Tropical Medicine, Department of Clinical Sciences<br>Devereux, Graham ; Liverpool School of Tropical Medicine, Department of Clinical Sciences<br>Obasi, Angela; Liverpool School of Tropical Medicine, Department of International Public Health<br>Mortimer, Kevin; Liverpool School of Tropical Medicine, Department of Clinical Sciences<br>Khan, Jahangir; Liverpool School of Tropical Medicine, Department of International Public Health; University of Gothenburg, School of Public Health and Community Medicine<br>Rylance, Jamie; Liverpool School of Tropical Medicine, Department of Clinical Sciences; Malawi-Liverpool-Wellcome Trust Clinical Research Programme, Lung Health Group<br>Niessen, Louis; Liverpool School of Tropical Medicine, Department of International Public Health ; Johns Hopkins University Bloomberg School of Public Health, Department of International Health |


| | |
|---|---|
| <b>Primary Subject Heading</b>: | Health economics |
| Secondary Subject Heading: | Epidemiology, Global health, Respiratory medicine |
| Keywords: | Respiratory infections < THORACIC MEDICINE, Economics < TROPICAL MEDICINE, EPIDEMIOLOGY, PUBLIC HEALTH |
| | |


SCHOLARONE™ Manuscripts

# Household food insecurity, maternal nutrition, environmental risks and infants' health outcomes: protocol of the IMPALA birth cohort study in Uganda

Zelalem G. Terfa[1, 3, *,‡], Rebecca Nantanda[1,4, *], Maia Lesosky[1,5], Graham Devereux[1], Angela Obasi[2], Kevin Mortimer[1], Jahangir Khan[2,6], Jamie Rylance[1,7, †], Louis W. Niessen[2,8, †], IMPALA Consortium

1. Department of Clinical Sciences, Liverpool School of Tropical Medicine, Liverpool, UK
2. Department of International Public Health, Liverpool School of Tropical Medicine, Liverpool, UK
3. College of Business and Economics, Addis Ababa University, Addis Ababa, Ethiopia
4. Makerere University Lung Institute, Makerere University College of Health Sciences, Kampala, Uganda
5. School of Public Health and Family Medicine, Faculty of Health Sciences, University of Cape Town, South Africa
6. School of Public Health and Community Medicine, University of Gothenburg, Sweden
7. Lung Health Group, Malawi Liverpool Wellcome Trust Clinical Research Programme, Queen Elizabeth Central Hospital, College of Medicine, P.O. BOX 30096, Chichiri, Blantyre, Malawi
8. Department of International Health, Johns Hopkins School of Public Health, Baltimore, Maryland, USA.

[*] equally contributed

[†] equally contributed

**Word count:** 3,828 words

‡ *Corresponding author: Email: Zelalem.Terfa@lstmed.ac.uk; Address: Pembroke Place, Liverpool L3 5QA UK*

**Abstract**

**Introduction:** In low-and middle-income countries (LMICs), food insecurity and undernutrition disproportionately affect women of reproductive age, infants, and young children. The disease burden from undernutrition in these vulnerable sections of societies remains a major concern in LMICs. Biomass fuel use for cooking is also common in LMICs. Empirical evidence from high income countries indicates that early life nutritional and environmental exposures and their effect on infant lung function are important, however data from Sub-Saharan Africa are scarce.

**Aim:** To estimate the association between infant lung function and household food insecurity, energy poverty, and maternal dietary diversity.

**Methods and analysis:** Pregnant women will be recruited in an existing Health and Demographic Surveillance Site in South-West Uganda. Household food insecurity, sources and uses of energy, economic measures and maternal dietary diversity will be collected during pregnancy and after birth. Primary health outcomes will be infant lung function determined by tidal breath flow and volume (TBFV) analysis at 6 -10 weeks of age. Infant weight and length will also be collected.

A household food consumption score and minimum dietary diversity for women (MDD-W) indicator will be constructed. The involved cost of dietary diversity (CoDD) will be estimated based on MDD-W. The association between household level and mothers' food access indicators and infant lung function will be evaluated using regression models. The multi-dimensional energy poverty index (MEPI) will be estimated and used as an indicator of households' environmental exposures. The association between household MEPI and infant lung function will be assessed using econometric models.

**Ethics and dissemination:** Ethical approvals have been obtained from Liverpool School of Tropical Medicine (18-059), the Uganda Virus Research Institute Ethics Committee (097/2018) and Uganda National Council for Science and Technology (SS 4846). Study results will be shared with participants, policy makers, other stakeholders and published in peer-reviewed journals.

**Study dates:** 1 April 2019 to 31 August 2021



For peer review only - http://bmjopen.bmj.com/site/about/guidelines.xhtml

**Strengths and limitations of this study**

- A key strength is the use of a birth cohort study design that allows prospective assessment of early life exposures and their association with infant lung function.
- We will generate novel data with the potential to inform much needed interventions to address important global health challenges of nutrition and adverse environmental exposures.
- The main limitation is the necessity for clinic-based recruitment of pregnant women for logistic reasons. Women who do not attend ANC will be systematically excluded. These women are likely the most disadvantaged and living in extreme poverty, this may represent an important selection bias. The study is located in only one district that is a longstanding surveillance may have resulted in the district being atypical. This could limit the generalisability of our findings.

For peer review only



For peer review only - http://bmjopen.bmj.com/site/about/guidelines.xhtml

## Introduction

In recent decades, despite the world has witnessed significant progress in poverty reduction, food insecurity and undernourishment are still pervasive in Sub-Saharan Africa (SSA). The proportion of undernourished people in SSA declined from 28.1% in 2000 to 20.6% in 2010, remained constant until 2015, and then increased to 22.8% in 2018 [1 2]. In the SSA region, Eastern Africa has the highest prevalence (30.8%) of undernourishment with more than 133 million affected [2]. The prevalence of total food insecurity is also highest in Eastern Africa, increasing from 58.2% in 2014 to 62.7 % in 2018 [2]. In Uganda specifically the proportion of undernourished population declined between 2004 and 2006 and then increased to 39% between 2014 and 2016 [1].

In low- and middle-income countries (LMICs) food insecurity and malnutrition disproportionately affect women of reproductive age, infants, and young children [3-6]. Women living in LMICs are particularly at risk of malnutrition during pregnancy and lactation due to increased nutritional requirements, socio-economic constraints, societal norms, high intensity of agricultural labour, and short interpregnancy intervals [7].

A child's nutritional status in utero and the postnatal period is inextricably linked to their mother's nutritional status [8]. Intergenerational effects increase infant mortality and have longer-term sequelae [4 9 10]. In Tanzania about 40% of mothers with under 2 year old didn't consume a diverse diet and 35% of children were stunted [11]. Household food insecurity is a risk factor for iron-deficiency anaemia in infants and toddlers [12], is associated with small birth size [13], and adverse infant health outcomes [14]. There is increasing interest in the early life origins and programming of chronic non-communicable diseases [8] such as the major chronic respiratory diseases of asthma and COPD [15]. In high income countries (HICs) there is a growing body of evidence indicating that sub-optimal in utero airway and parenchymal lung development are important risk factors for childhood asthma and probably COPD [16 17]. Maternal nutrition, smoking and exposure to air pollutants have been shown to be associated with reduced infant lung function, changes in neonatal immune and airway epithelial cell function and increased risk of childhood wheezing illness [18-21].

Poorer households, particularly in low-income countries tend to have lower-quality diets and select foods of lower nutritional value and lower-quality diets [22 23]. Household-level strategies to cope shortfalls in food availability tend to further reduce both quality and quantity [24], and food prices can significantly moderate this effect [25]. Food price rises also reduce dietary diversity [26] and consequently nutritional outcomes [23].

Household air pollution exposure is a predisposing factor for adverse health outcomes in children. In Ghana, pre-natal exposure to air pollution is associated with reduced tidal volumes and higher minute ventilation and airway inflammation in 5 week old infants [27]. In Nepal biomass smoke exposure was associated with lung function deficits in adults [28]. In India poverty is strongly associated with reduced paediatric lung function, in addition to body mass index, tobacco exposure and biomass use [29]. . Household multidimensional energy poverty index , a measure of households deprivation from clean energy source [30], can be used as a proxy to assess the association between household air pollution and lung health outcomes of young infants. Though varying dimensions of energy poverty indicators



For peer review only - http://bmjopen.bmj.com/site/about/guidelines.xhtml

have been used in different contexts, studies have shown this parameter is associated with respiratory illness and other health outcome in infants and children [31 32 33].

Empirical evidence of association between early life nutritional and environmental exposures with infant lung function and nutritional outcomes is important to support policy makers in setting priorities to improve early childhood development and identify intervention mechanisms. Empirical evidence on the effects of in utero nutritional and environmental exposures and infant health outcome are almost exclusively based in higher income countries [9 18 19 21 34]. In LMICs few studies have focused on in utero nutritional exposures and non-communicable diseases [11-13]. No studies from Sub-Saharan Africa have examined the association of households' nutritional and environmental exposure on the respiratory health outcome of infants, particularly infant and maternal lung function. Inequalities in infant lung health outcomes in relation to household socioeconomic status are also unknown in the region. The study presented here will generate a unique body of quantitative evidence in a low-income setting pertaining to the prevalence and distribution of food insecurity, dietary diversity among pregnant/postnatal women, together with cost of dietary diversity (CoDD), energy poverty and its association with infant lung function and nutritional outcomes.

## Study goal and objectives

The study aims to estimate the association of households' food insecurity, mothers' dietary diversity, and energy poverty with infant lung function and nutritional outcomes. The specific objectives are as follows:

1. To estimate food insecurity at household level, dietary diversity of pregnant women, and related cost of dietary diversity (CoDD).
2. To assess the effect of household food insecurity and women's dietary diversity during pregnancy and after birth on infant's lung function and nutritional outcomes.
3. To estimate the association between the household energy poverty of pregnant women and their socio-economic status with the lung function of infants and mothers.

## Methods

## Study design

The study will use a prospective cohort design to measure exposures and outcomes among pregnant women and their infants. Data on household food consumption, energy sources, maternal demographics and dietary diversity will be collected during pregnancy and 6 to 10 weeks after birth. Infant lung function and other health outcome data will also be collected at 6-10 weeks of age. Data collection at 6-10 weeks will enable comparisons with other similar studies[21] and is conveniently the time mothers take their infants to clinics for vaccination.



For peer review only - http://bmjopen.bmj.com/site/about/guidelines.xhtml

**Study setting**

This study, is part of a larger, multi-country research programme, the International Multidisciplinary Programme to Address Lung Health and TB in Africa (IMPALA) [35]. The study will be conducted in the General Population Cohort (GPC) which is a Health and Demographic Surveillance Site (HDSS) in Kyamulibwa sub-county of Kalungu District in South-Western Uganda. The GPC is a population-based open cohort study, established in 1989 by the UK Medical Research Council in collaboration with the Uganda Virus Research Institute to examine trends in the prevalence and incidence of HIV and its determinants in rural south-Western Uganda[36] . The HDSS comprises 25 rural villages, and a small township of Kyamulibwa Town Council. Annual census and medical surveys have been conducted in this population since 1989 [37] .

The main economic activity in the site is agriculture characterized by small farms of bananas, coffee, legumes, vegetables, cassava, and potatoes. There is also small-scale cattle, goat and pig farming. A few residents are engaged in small scale trading, selling coffee, food crops and fish. The education level of inhabitants is generally low with only a third attaining secondary level schooling. There are no special health, economic and social support programmes in the HDSS. The livelihoods and challenges of the residents are generally considered to be typical of rural populations in Uganda.

Five health facilities serve the population with basic medical care, of which three facilities offer maternity services, antenatal care, deliveries, and postnatal care. The HDSS has a pregnancy and birth registration system whereby pregnant women are registered and followed up until delivery to document birth outcomes. Births are notified to the HDSS administration within 24-48 hours. In Uganda, 97% of pregnant women attend at least one antenatal clinic visit [38]. The existing systems within the HDSS are favourable for conducting cohort studies, including the established birth notification and registration system, periodic censuses, and medical surveys. This facilitates the tracking of women through pregnancy, delivery, and post-partum, minimizing loss to follow up, and allowing future studies.

**Study population**

The study will recruit pregnant women, their infants, and households.

**Sampling strategy**

This cohort study will be conducted at the three health facilities that offer maternity services in the HDSS. Recruitment will be preceded by a comprehensive programme of community sensitisation. Pregnant women will be identified whilst attending the antenatal clinics. Consecutive pregnant women will be approached to participate in the study. Written informed consent will be obtained from all participants.

Same day recruitment will be performed if potential participants wish, to minimise inconvenience and costs of repeat travel for participants. However, if potential participants wish to take time to consider participation and/or discuss with members of their family, this will be facilitated.



For peer review only - http://bmjopen.bmj.com/site/about/guidelines.xhtml

### Sample size calculation

The sample size estimation is based on estimation of the association between the primary exposures (maternal dietary diversity and household food security) with infant lung function (minute ventilation ml/min). The primary outcome will be infant minute ventilation, and study power is based on a birth cohort study that related air pollution during pregnancy to infant lung function in which maternal air pollution (PM10) exposure measurements were significantly associated with minute ventilation in infants aged 6-10 weeks, and reported a mean (SD) minute ventilation of 1401 (242) ml/min [21].

Maternal dietary diversity is measured by a dichotomous indicator whilst household food insecurity is a three-scale categorical variable. The proposed study will have 80% power to detect a 5% difference in minute volume between high and low maternal dietary diversity groups and a 6% difference across the thirds of household food insecurity at the 5% level of significance. With a minimum sample size of 360 (complete data), we will have at least 80% power to estimate moderate effect sizes (OR ≥ 1.5) under a logistic regression model, and >90% power to estimate differences of 5% in continuous infant lung function outcomes with 95% confidence, under a linear regression model. We therefore aim to recruit 560 pregnant women to compensate for an anticipated 10% loss to follow up, a 5% rate of miscarriages, still births, and neonatal deaths, and failure to obtain valid lung function test results in 25% of infants.

### Eligibility criteria

*Pregnant women:* All pregnant women, irrespective of gestational age, presenting for ANC services at the selected health facilities and residing within the HDSS will be eligible to participate. Women who will not be available throughout the entire study period (such as those intending to go to another area for delivery of their babies or other reasons) and/or unwilling to participate in the study will be excluded.

*Infants:* All singleton infants will be eligible. It will not be possible to conduct lung function tests for infants with congenital abnormalities of the upper airway for obvious reasons. Therefore, these infants (likely to be very few) will be excluded at analysis of the primary outcome (minute ventilation).

### Data collection and analysis methods

The data collection process will follow a number of steps. The data will be collected at four-time points:

- Time-point 1: during the antenatal clinic, recruitment point.
- Time-point 2: home visit to the pregnant woman households, one weeks after recruitment.
- Time-point 3: clinic lung function testing for mothers and their infants, 6 to 10 weeks after birth.
- Time-point 4: home visit the women's household during 6 to 10 weeks after birth.



For peer review only - http://bmjopen.bmj.com/site/about/guidelines.xhtml

A summary of participant contacts and required data to be collected at each of the four-time points is presented in flowchart (Figure 1). Details of data collection and analytical methods are presented under each of the sub-study topics. There will be three sub-studies as listed below.

**(Figure 1 about here!)**

**Study 1: Estimate food insecurity at household level, dietary diversity of pregnant women together with cost of dietary diversity (CoDD)**

The main aim of this sub-study is to characterize households' food insecurity and dietary diversity for participating pregnant women. Closely related to dietary diversity of pregnant women, the cost of dietary diversity (CoDD) will be estimated. Socioeconomic determinants of household food insecurity and women's dietary diversity will also be identified.

**Data collection**

Household level food consumption and expenditure data will be collected using the World Bank's living standard questionnaire that has been used in many African countries, including Uganda. Consumption questionnaire will be mapped with Food consumption score (FCS) food items and groups. The food consumption score (FCS) is a validated tool to assess household level food insecurity. It combines data on dietary diversity and food frequency using 7 days recall data [39]. The main advantage of this tool is that it captures dietary diversity and household food access. It also enables an understanding of the usual consumption behaviour of households [40 41]. FCS will be used to assess household level food insecurity during pregnancy (and after birth). Data on household's food insecurity coping strategies will also be collected. In addition, separate data will be collected using the Minimum Dietary Diversity for Women(MDD-W)questionnaire, a validated tool, [3] to assess pregnant women's dietary diversity. We will do adaptation work for the MDD-W questionnaire based on local context. Adaptation of the MDD-W questionnaire is helpful for adequate listing of locally available food items, contents of mixed food and food items that are consumed in trivial amounts and to reflect cultural norms, vocabulary and usage of words and phrases that will be easily understood.

Nationally representative monthly food price data will be obtained from Uganda Bureau of Statistics to estimate the cost of dietary diversity (CoDD) for women of reproductive age. CoDD is a price index defined around the MDD-W. It is an indicator that provides the least expensive way of meeting the MDD-W [42].

Anthropometric (weight, height, and Mid Upper Arm Circumference-MUAC) and demographic data (including age) will be collected from pregnant women. The same anthropometric measuring equipment will be used across the clinics. As described above women's anthropometric measurements will be taken at clinics during pregnancy check-up



For peer review only - http://bmjopen.bmj.com/site/about/guidelines.xhtml

visits. Data on household asset ownership will be collected. These data will include education, access to land, livestock holdings, type of crops grown, farm and other household assets, non-farm activities of the household. This data will be used to construct household asset index.

**Data Analysis:** Data will be analysed using both descriptive and multivariable regression models. The household level food consumption score (FCS) will be obtained from frequency of consumption of each food group in the household and by assigning standard weight for each food groups. This score will be used to categorize households into three levels of food insecurity status: poor, borderline, and acceptable following the standard cut-off points [39 40 43 44]. To construct the MDD-W indicator, food groups and sub-food groups will be aggregated into 10 MDD-W food groups. Woman's dietary diversity will be categorised into two based on standard threshold: scoring ≥ 5 and below the threshold [3 45 46]. The CoDD will be estimated based on rank order optimization based on food prices within group as described by Masters, et al. [42]. In this technique, cheapest food items will be selected from each of the ten food groups then the five most cheapest food items will be selected. This identifies the cheapest way to achieve MDD-W. This will be done on monthly basis and will be linked with monthly dietary diversity of pregnant women.

Associations of household level food insecurity and women's dietary diversity will be assessed using parametric or non-parametric statistical tests depending on distribution of the variables. Determinants of household food insecurity, and dietary diversity amongst women will be assessed using discrete choice based regression models. The household level food insecurity, the dependent variable, is an ordered categorical variable. This suggests the natural choice will be the ordered logit (or probit) regression model. Maternal dietary diversity takes dichotomous variables. Therefore, a multivariable logit (or probit) regression model will be used to identify determinants of dietary diversity in women. Principal Component Analysis (PCA) will be used to construct households socioeconomic status index, using asset data, to categorize households into socioeconomic quintiles and the method is explained elsewhere [47 48]. Then distribution of household food insecurity and women's dietary diversity across socioeconomic status will be assessed.

**Study 2: Assessing the effect of household food insecurity and women's dietary diversity on infant's lung function and nutritional outcomes**

The dynamics of household food insecurity during pregnancy and after birth and its impacts on lung function and the nutritional outcomes measures (birth weight and length) of infants will be assessed. Changes in mothers' dietary diversity during pregnancy and after birth will be thoroughly assessed.

**Data collection**

Data collection from study 1 will be repeated in the same individuals 6 to 10 weeks after birth. In addition, infant anthropometric measures (weight and length) will be collected. We will also collect data on method of delivery. Preterm babies will also be identified during data collection. Maternal lung function (spirometry) and anthropometric data (weight and height) will also be collected. In addition, infant lung function will be measured. Infant lung function testing will be performed during quiet unsedated breathing using the Tidal Breath



For peer review only - http://bmjopen.bmj.com/site/about/guidelines.xhtml

Analysis (TBA) method performed by clinical sciences research team in IMPALA programme. Tidal breathing will be taken as the natural physiological state of undisturbed regular breathing, according to the European Respiratory Society (ERS)/American Thoracic Society (ATS) standards of infant lung function testing [49] . With the sleeping infant is a supine position and neck slightly extended, a face mask will be carefully and gently applied onto his/her mouth and nose. The infant will be given 2-5 minutes to adapt to the mask before actual measurement of the tidal breaths starts. Tidal breathing will be recorded for a total of 10 minutes. This time is deemed sufficient to obtain valid information regarding the lung functioning. Tidal breathing measures of Minute ventilation (MV), tidal volume (VT), respiratory rate and expiratory flow ratios will be collected using the Exhalyser D with ultrasonic flow metre (Ecomedics, Duernton, Switzerland). The tests will be conducted using the Exhalyser D with ultrasonic flow metre (Ecomedics, Duernton, Switzerland) and interpreted using Spiroware version 3.2.

**Data Analysis**

Household level food insecurity, and maternal dietary diversity will be measured using the techniques discussed under study 1. The dynamics in household food insecurity and women's dietary will be analysed using descriptive statistics.

The association between household level and mothers' food access indicators and infant lung function (measured by using MV, VT, RR, tPEF:tE) and nutritional outcomes (weight-for-age and length-for-age) of infants will be evaluated using appropriate econometric models. We propose to use multiple linear regression model to estimate the association.

Household socioeconomic status will be categorized based on asset index. Principal Component Analysis (PCA) will be used to construct household socioeconomic status index. Distributions of lung function and other health outcome across socioeconomic status of households will be examined using a concentration curve [50 51]. The concentration curve gives a more complete picture of socioeconomic inequality in health outcomes. However, it does not give a measure of the magnitude of inequality [47]. Therefore, concentration index, which quantifies the degree of socioeconomic related inequality in a health variable [50-52] will be used to measure the degree of socioeconomic-related inequality in infant's and mother's lung function and other health outcomes.

These analyses will lead us to identify socioeconomic determinants of infants' lung function and other health outcome (nutritional status of infants).

**Study 3: Estimate the association of household energy poverty and their socio-economic status with lung function of infants and mothers**

This sub-study aims to assess association between household energy poverty and the lung function of infants and their mothers. The sources of energy used by households, available/alternative energy sources, and households' access to alternative energy sources and implication for risk of respiratory diseases will be explored. Inequalities in infant lung



For peer review only - http://bmjopen.bmj.com/site/about/guidelines.xhtml

function and nutritional outcomes across socioeconomic status of households will be explored.

**Data collection**

Data on household energy source and use will be collected. Attention will be given to energy sources used by households, access to different sources of energy, and energy by category of use. We will also collect data on ownership of appliances, entertainment and communication equipment that rely on access to electricity. The lung function of infants will be measured at 6-10 weeks after birth using methods described under sub-study 2. The lung function of the mothers will be assessed using Spirometry, performed according to the European Respiratory Society (ERS)/American Thoracic Society (ATS) standards for Spirometry [53]. Up to eight forced expiratory manoeuvres will be recorded using EasyOn spirometer (ndd, Switzerland), with quality control performed by an experienced clinician. Additional external review will take place for 10% of traces by a respiratory specialist.

**Data analysis**

Multi-dimensional energy poverty index (MEPI) will be estimated from household energy use and sources data. The MEPI captures and evaluates a set of energy deprivations that affect households. Following Nussbaumer, et al. [30], the MEPI that is composed of five dimensions representing basic energy services and six indicators of these dimensions will be estimated. Essentially, a household is identified as energy poor if the respective set of deprivation exceeds a pre-defined threshold. However, there is no standard and agreed threshold. We will categorize households into quintiles based on MEPI and assess the distribution of infants' and women's lung function along the quintiles. Inequalities in nutritional outcome of infants will be assess using method discussed under sub-study 2. Association between energy poverty and infants' lung function will be evaluated using multivariable regression models.

**Patient and public involvement**

We appreciate movement towards involvement of patients and the public as co-producers of research. The communities and community leaders were involved in community-level sessions. Patient representatives were not directly involved in the development of this research project. We will seriously consider this in future studies.

**Discussion**

The study will provide new and valuable quantitative empirical evidence on the relationships and interactions between household level food insecurity, maternal dietary diversity, energy poverty, and infant lung function and nutritional outcomes in a low-income setting. We are not aware of other any similar studies in Sub-Sahara Africa. This evidence is critical for policy makers to make informed decisions in identifying and prioritizing interventions to address the burden and transgenerational consequences of maternal malnutrition during pregnancy. It will also provide a better understanding of public health implications of energy poverty and indoor air pollution at household level.

For peer review only

A key strength of this study is the use of a birth cohort study design which allows prospective assessment of early life exposures and their association with infant health outcomes. The necessity for clinic-based recruitment of pregnant women for logistic reasons (availability of lung function testing equipment) will result in women who do not attend ANC being systematically excluded. These women are likely to be the most disadvantaged and living in extreme poverty, this may represent an important selection bias. However, ANC uptake in Uganda is high [38] which should limit the size of this bias. The fact that our study is located in only one district and that longstanding surveillance [54] may have caused that district to be atypical in other ways, could limit the generalisability of our findings.

However, improving infant and maternal nutrition is a key global health priority. It is also increasingly recognised that poor lung function is associated with increased respiratory and non-respiratory morbidity and mortality across the life course [54]. Our prospective study will generate novel data with the potential to inform much needed interventions to address these important global health challenges.

**Figure legend/caption:**

**Figure 1** Flowchart for summary of data collection process and required data at each of data collection steps.

**Ethics and dissemination**

The study received ethical approval from Liverpool School of Tropical Medicine (LSTM) (18-059), the Uganda Virus Research Institute Ethics Committee (097/2018) and Uganda National Council for Science and Technology (SS 4846). Findings from this study will be disseminated through peer-reviewed publications and conference presentations. We also intend to disseminate the findings using other platforms including community feedback meetings and engagement with national policy makers and health care planners.

**Funding:** This research was commissioned by the National Institute of Health Research using Official Development Assistance (ODA) funding (IMPALA grant number 16/136/35). The views expressed in this publication are those of the author(s) and not necessarily those of the NHS, the National Institute for Health Research or the Department of Health.

**Competing interest statement:** there are no competing interests for any author.

**First and senior Authors:** ZGT and RN equally contributed to this paper and are joint first authors. LWN and JR are joint senior authors

**Contributors:** ZGT, RN, JR, JK, LWN conceptualized and designed the study. ZGT and RN wrote the protocol and drafted the protocol manuscript with guidance from LWN, JR, and JK. GD helped during protocol development and contributed to methodological, dietary and ethical aspects of the study. ML helped with methodological aspect of the study, including



For peer review only - http://bmjopen.bmj.com/site/about/guidelines.xhtml

sampling design and sample calculations. AO critically reviewed the protocol manuscript and helped during initial stage of designing the study. KM is director of IMPALA and played key role during early conceptualization of the study. IMPALA consortium members provided useful comments to protocol manuscript.

**Collaborators:** On behalf of The NIHR International Multidisciplinary Programme to Address Lung Health and TB in Africa (IMPALA) Consortium, which in addition to the above named authors comprises: Emmanuel Addo-Yobo, Brian Allwood, Hastings Banda, Imelda Bates, Amsalu Binegdie, Asma El Sony, Adegoke Falade, Bertrand Mbatchou, Hellen Meme, Beatrice Mutayoba, Muhwa Jeremiah Chakaya, Nyanda Elias Ntinginya, S Bertel Squire, Miriam Taegtmeyer, Rachel Tolhurst, William Worodria, Heather Zar, Eliya Zulu, Lindsay Zurba.

For peer review only



For peer review only - http://bmjopen.bmj.com/site/about/guidelines.xhtml

## References


1. FAO. Regional Overview of Food Security and Nutrition in Africa 2017. The food security and nutrition–conflict nexus: building resiliencefor food security, nutrition and peace. Accra, 2017.
2. FAO, IFAD, UNICEF, et al. The State of Food Security and Nutrition in the World 2019. Safeguarding against economic slowdowns and downturns. Rome, FAO., 2019.
3. FAO, FHI 360. Minimum Dietary Diversity for Women: A Guide for Measurement. Rome:: FAO, 2016.
4. Lartey A. Maternal and child nutrition in Sub-Saharan Africa: challenges and interventions. *Proceedings of the Nutrition Society* 2008;67(1):105-08. doi: 10.1017/S0029665108006083 [published Online First: 2008/01/30]
5. Allen LH. Multiple micronutrients in pregnancy and lactation: an overview. *The American Journal of Clinical Nutrition* 2005;81(5):1206S-12S. doi: 10.1093/ajcn/81.5.1206
6. Black RE, Allen LH, Bhutta ZA, et al. Maternal and child undernutrition: global and regional exposures and health consequences. *The Lancet* 2008;371(9608):243-60. doi: https://doi.org/10.1016/S0140-6736(07)61690-0
7. Lee SE, Talegawkar SA, Merialdi M, et al. Dietary intakes of women during pregnancy in low- and middle-income countries. *Public Health Nutrition* 2013;16(8):1340-53. doi: 10.1017/S1368980012004417 [published Online First: 2012/10/09]
8. Christian P, Stewart CP. Maternal Micronutrient Deficiency, Fetal Development, and the Risk of Chronic Disease. *The Journal of Nutrition* 2010;140(3):437-45. doi: 10.3945/jn.109.116327
9. Roseboom TJ, van der Meulen JHP, Ravelli ACJ, et al. Effects of prenatal exposure to the Dutch famine on adult disease in later life: an overview. *Molecular and Cellular Endocrinology* 2001;185(1):93-98. doi: https://doi.org/10.1016/S0303-7207(01)00721-3
10. Victora CG, Adair L, Fall C, et al. Maternal and child undernutrition: consequences for adult health and human capital. *The Lancet* 2008;371(9609):340-57. doi: 10.1016/S0140-6736(07)61692-4
11. Huang M, Sudfeld C, Ismail A, et al. Maternal Dietary Diversity and Growth of Children Under 24 Months of Age in Rural Dodoma, Tanzania. *Food and Nutrition Bulletin* 2018:0379572118761682. doi: 10.1177/0379572118761682
12. Park K, Kersey M, Geppert J, et al. Household food insecurity is a risk factor for iron-deficiency anaemia in a multi-ethnic, low-income sample of infants and toddlers. *Public Health Nutrition* 2009;12(11):2120-28. doi: 10.1017/S1368980009005540 [published Online First: 2009/05/01]
13. Chowdhury M, Dibley MJ, Alam A, et al. Household Food Security and Birth Size of Infants: Analysis of the Bangladesh Demographic and Health Survey 2011. *Current Developments in Nutrition* 2018;2(3):nzy003-nzy03. doi: 10.1093/cdn/nzy003
14. Cook JT, Frank DA, Berkowitz C, et al. Food Insecurity Is Associated with Adverse Health Outcomes among Human Infants and Toddlers. *The Journal of Nutrition* 2004;134(6):1432-38. doi: 10.1093/jn/134.6.1432
15. Bousquet J, Kaltaev N. Global surveillance, prevention and control of chronic respiratory diseases : a comprehensive approach / edited by Jean Bousquet and Nikolai Khaltaev. Geneva: World Health Organization, 2007.
16. Stocks J, Hislop A, Sonnappa S. Early lung development: lifelong effect on respiratory health and disease. *The Lancet Respiratory Medicine* 2013;1(9):728-42. doi: 10.1016/S2213-2600(13)70118-8
17. Bush A. Lung Development and Aging. *Ann Am Thorac Soc* 2016;13 Suppl 5:S438-s46. doi: 10.1513/AnnalsATS.201602-112AW [published Online First: 2016/12/23]




For peer review only - http://bmjopen.bmj.com/site/about/guidelines.xhtml

18. Miller DR, Turner SW, Spiteri-Cornish D, et al. Maternal vitamin D and E intakes during early pregnancy are associated with airway epithelial cell responses in neonates. *Clinical & Experimental Allergy* 2015;45(5):920-27. doi: https://doi.org/10.1111/cea.12490
19. Devereux G, Barker RN, Seaton A. Antenatal determinants of neonatal immune responses to allergens. *Clin Exp Allergy* 2002;32(1):43-50. doi: 10.1046/j.0022-0477.2001.01267.x [published Online First: 2002/05/11]
20. STOCKS J, DEZATEUX C. The effect of parental smoking on lung function and development during infancy. *Respirology* 2003;8(3):266-85. doi: https://doi.org/10.1046/j.1440-1843.2003.00478.x
21. Latzin P, Roosli M, Huss A, et al. Air pollution during pregnancy and lung function in newborns: a birth cohort study. *The European respiratory journal* 2009;33(3):594-603. doi: 10.1183/09031936.00084008 [published Online First: 2008/11/18]
22. Darmon N, Drewnowski A. Contribution of food prices and diet cost to socioeconomic disparities in diet quality and health: a systematic review and analysis. *Nutrition Reviews* 2015;73(10):643-60. doi: 10.1093/nutrit/nuv027
23. Miller V, Yusuf S, Chow CK, et al. Availability, affordability, and consumption of fruits and vegetables in 18 countries across income levels: findings from the Prospective Urban Rural Epidemiology (PURE) study. *The Lancet Global Health* 2016;4(10):e695-e703. doi: https://doi.org/10.1016/S2214-109X(16)30186-3
24. Farzana FD, Rahman AS, Sultana S, et al. Coping strategies related to food insecurity at the household level in Bangladesh. *PLOS ONE* 2017;12(4):e0171411. doi: 10.1371/journal.pone.0171411
25. Green R, Cornelsen L, Dangour AD, et al. The effect of rising food prices on food consumption: systematic review with meta-regression. *BMJ : British Medical Journal* 2013;346:f3703. doi: 10.1136/bmj.f3703
26. Brinkman H-J, de Pee S, Sanogo I, et al. High Food Prices and the Global Financial Crisis Have Reduced Access to Nutritious Food and Worsened Nutritional Status and Health. *The Journal of Nutrition* 2009;140(1):153S-61S. doi: 10.3945/jn.109.110767
27. Lee AG, Kaali S, Quinn A, et al. Prenatal Household Air Pollution Is Associated with Impaired Infant Lung Function with Sex-Specific Effects. Evidence from GRAPHS, a Cluster Randomized Cookstove Intervention Trial. *Am J Respir Crit Care Med* 2019;199(6):738-46. doi: 10.1164/rccm.201804-0694OC [published Online First: 2018/09/27]
28. Kurmi OP, Devereux GS, Smith WCS, et al. Reduced lung function due to biomass smoke exposure in young adults in rural Nepal. *European Respiratory Journal* 2013;41(1):25-30. doi: 10.1183/09031936.00220511
29. Sonnappa S, Lum S, Kirkby J, et al. Disparities in Pulmonary Function in Healthy Children across the Indian Urban–Rural Continuum. *American Journal of Respiratory and Critical Care Medicine* 2015;191(1):79-86. doi: 10.1164/rccm.201406-1049OC
30. Nussbaumer P, Bazilian M, Modi V. Measuring energy poverty: Focusing on what matters. *Renewable and Sustainable Energy Reviews* 2012;16(1):231-43. doi: https://doi.org/10.1016/j.rser.2011.07.150
31. Mohan G. Young, poor, and sick: The public health threat of energy poverty for children in Ireland. *Energy Research & Social Science* 2021;71:101822. doi: https://doi.org/10.1016/j.erss.2020.101822
32. Nawaz S. Energy poverty, climate shocks, and health deprivations. *Energy Economics* 2021;100:105338. doi: https://doi.org/10.1016/j.eneco.2021.105338
33. Banerjee R, Mishra V, Maruta AA. Energy poverty, health and education outcomes: Evidence from the developing world. *Energy Economics* 2021;101:105447. doi: https://doi.org/10.1016/j.eneco.2021.105447



For peer review only - http://bmjopen.bmj.com/site/about/guidelines.xhtml

34. Bisgaard H, Loland L, Holst KK, et al. Prenatal determinants of neonatal lung function in high-risk newborns. *The Journal of allergy and clinical immunology* 2009;123(3):651-7, 57.e1-4. doi: 10.1016/j.jaci.2008.11.036 [published Online First: 2009/01/21]
35. Ding Y, Pulford J, Bates I. Practical actions for fostering cross-disciplinary global health research: lessons from a narrative literature review. *BMJ Global Health* 2020;5(4):e002293. doi: 10.1136/bmjgh-2020-002293
36. Asiki G, on behalf of the GPCt, Murphy G, et al. The general population cohort in rural south-western Uganda: a platform for communicable and non-communicable disease studies. *International Journal of Epidemiology* 2013;42(1):129-41. doi: 10.1093/ije/dys234
37. Asiki G, Murphy GAV, Baisley K, et al. Prevalence of Dyslipidaemia and Associated Risk Factors in a Rural Population in South-Western Uganda: A Community Based Survey. *PLOS ONE* 2015;10(5):e0126166. doi: 10.1371/journal.pone.0126166
38. UBOS, ICF. Uganda Bureau of Statistics (UBOS) and ICF. 2018. Uganda Demographic and Health Survey 2016. Kampala, Uganda and Rockville, Maryland, USA: UBOS and ICF., 2018.
39. WFP. World Food Programme. Food consumption analysis: calculation and use of the Food Consumption Score in food consumption and food security analysis. Technical Guidance Sheet. Rome. 2008
40. Jones AD, Ngure FM, Pelto G, et al. What Are We Assessing When We Measure Food Security? A Compendium and Review of Current Metrics. *Advances in Nutrition* 2013;4(5):481-505. doi: 10.3945/an.113.004119
41. Maxwell D, Vaitla B, Coates J. How do indicators of household food insecurity measure up? An empirical comparison from Ethiopia. *Food Policy* 2014;47:107-16. doi: https://doi.org/10.1016/j.foodpol.2014.04.003
42. Masters WA, Bai Y, Herforth A, et al. Measuring the Affordability of Nutritious Diets in Africa: Price Indexes for Diet Diversity and the Cost of Nutrient Adequacy. *American Journal of Agricultural Economics* 2018;100(5):1285-301. doi: 10.1093/ajae/aay059
43. Wiesmann D, Bassett L, Benson T, et al. Validation of the World Food Programme's Food Consumption Score and Alternative Indicators of Household Food Security: Intl Food Policy Res Inst 2009.
44. Leroy JL, Ruel M, Frongillo EA, et al. Measuring the Food Access Dimension of Food Security:A Critical Review and Mapping of Indicators. *Food and Nutrition Bulletin* 2015;36(2):167-95. doi: 10.1177/0379572115587274
45. Arimond M, Wiesmann D, Becquey E, et al. Simple Food Group Diversity Indicators Predict Micronutrient Adequacy of Women's Diets in 5 Diverse, Resource-Poor Settings. *The Journal of Nutrition* 2010;140(11):2059S-69S. doi: 10.3945/jn.110.123414
46. Saha KK, Frongillo EA, Alam DS, et al. Household food security is associated with growth of infants and young children in rural Bangladesh. *Public Health Nutrition* 2009;12(9):1556-62. doi: 10.1017/S1368980009004765 [published Online First: 2009/09/01]
47. O'Donnell O, van Doorslaer E, Wagstaff A, et al. Analyzing health equity using household survey data: a guide to techniques and their implementation. Washington, D.C: World Bank Publications 2008.
48. Vyas S, Kumaranayake L. Constructing socio-economic status indices: how to use principal components analysis. *Health Policy and Planning* 2006;21(6):459-68. doi: 10.1093/heapol/czl029
49. Bates JH, Schmalisch G, Filbrun D, et al. Tidal breath analysis for infant pulmonary function testing. ERS/ATS Task Force on Standards for Infant Respiratory Function Testing. European Respiratory Society/American Thoracic Society. *The European respiratory journal* 2000;16(6):1180-92. doi: 10.1034/j.1399-3003.2000.16f26.x [published Online First: 2001/04/09]



For peer review only - http://bmjopen.bmj.com/site/about/guidelines.xhtml

50. Kakwani N, Wagstaff A, van Doorslaer E. Socioeconomic inequalities in health: Measurement, computation, and statistical inference. *Journal of Econometrics* 1997;77(1):87-103. doi: https://doi.org/10.1016/S0304-4076(96)01807-6
51. Wagstaff A, Paci P, van Doorslaer E. On the measurement of inequalities in health. *Social Science & Medicine* 1991;33(5):545-57. doi: https://doi.org/10.1016/0277-9536(91)90212-U
52. Wagstaff A, van Doorslaer E, Watanabe N. On decomposing the causes of health sector inequalities with an application to malnutrition inequalities in Vietnam. *Journal of Econometrics* 2003;112(1):207-23. doi: https://doi.org/10.1016/S0304-4076(02)00161-6
53. Miller MR, Hankinson J, Brusasco V, et al. Standardisation of spirometry. *European Respiratory Journal* 2005;26(2):319-38. doi: 10.1183/09031936.05.00034805
54. Agustí A, Noell G, Brugada J, et al. Lung function in early adulthood and health in later life: a transgenerational cohort analysis. *Lancet Respir Med* 2017;5(12):935-45. doi: 10.1016/s2213-2600(17)30434-4 [published Online First: 2017/11/19]

For peer review only



For peer review only - http://bmjopen.bmj.com/site/about/guidelines.xhtml

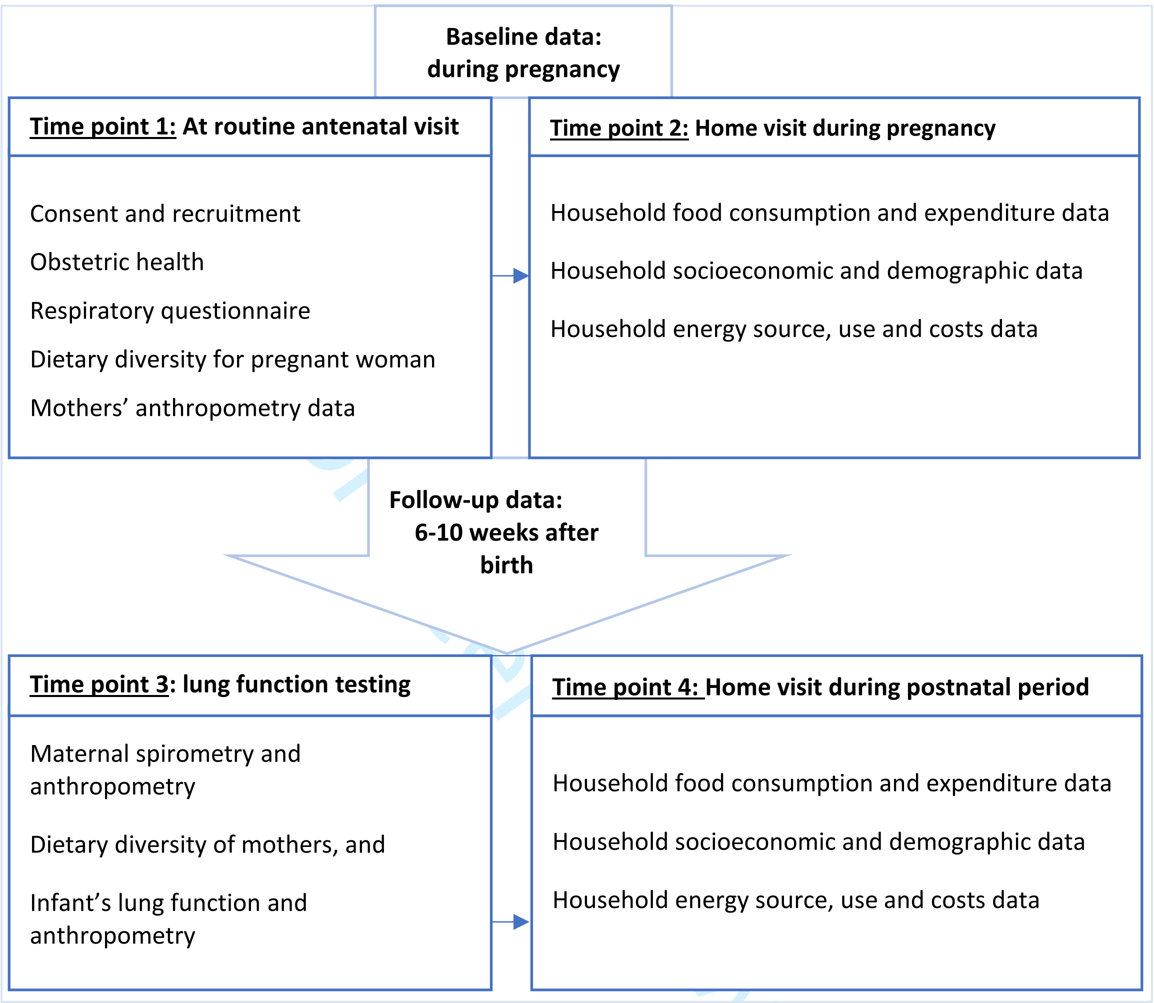


**Figure 1** Flowchart for summary of data collection process and required data at each of data collection steps.
